# Urbanization and Its Environmental Impact in Ceredigion County, Wales: A 20-Year Remote Sensing and GIS-Based Assessment (2003–2023)

**DOI:** 10.3390/s25175332

**Published:** 2025-08-27

**Authors:** Muhammad Waqar Younis, Edore Akpokodje, Syeda Fizzah Jilani

**Affiliations:** 1 Department of Computer Science, Aberystwyth University, Aberystwyth SY23 3DB, UK; eta@aber.ac.uk; 2 Department of Physics, Aberystwyth University, Aberystwyth SY23 3BZ, UK; sfj7@aber.ac.uk

**Keywords:** urbanization, land surface temperature (LST), urban heat island (UHI), remote sensing (RS), GIS, spectral indices (NDVI, NDWI, NDBI, NDBaI), sustainable urban planning

## Abstract

Urbanization is a dominant force reshaping human settlements, driving socio-economic development while also causing significant environmental challenges. With over 56% of the world’s population now residing in urban areas—a figure expected to rise to two-thirds by 2050—land use changes are accelerating rapidly. The conversion of natural landscapes into impervious surfaces such as concrete and asphalt intensifies the Urban Heat Island (UHI) effect, raises urban temperatures, and strains local ecosystems. This study investigates land use and landscape changes in Ceredigion County, UK, utilizing remote sensing and GIS techniques to analyze urbanization impacts over two decades (2003–2023). Results indicate significant urban expansion of approximately 122 km^2^, predominantly at the expense of agricultural and forested areas, leading to vegetation loss and changes in water availability. County-wide mean land surface temperature (LST) increased from 21.4 °C in 2003 to 23.65 °C in 2023, with urban areas recording higher values around 27.1 °C, reflecting a strong UHI effect. Spectral indices (NDVI, NDWI, NDBI, and NDBaI) reveal that urban sprawl adversely affects vegetation health, water resources, and land surfaces. The Urban Thermal Field Variance Index (UTFVI) further highlights areas experiencing thermal discomfort. Additionally, machine learning models, including Linear Regression and Random Forest, were employed to forecast future LST trends, projecting urban LST values to potentially reach approximately 27.4 °C by 2030. These findings underscore the urgent need for sustainable urban planning, reforestation, and climate adaptation strategies to mitigate the environmental impacts of rapid urban growth and ensure the resilience of both human and ecological systems.

## 1. Introduction

Urbanization represents one of the most significant transformation phenomena influencing human settlements and accelerating socio-economic development in recent decades [1]. Today, 56 percent of the world’s population lives in cities. Cities and city regions are centers of migration and human use, and their populations are critical to Earth’s starving biosphere. The United Nations predicts that this number will rise to between 5 to 5.7 billion people, which will represent about 70% of the world’s population. This rapid urban expansion is accompanied by changes in land use patterns, which include the conversion of natural landscapes, including vegetation and water bodies, into impermeable surfaces such as built and paved surfaces [2,3,4]. Such dramatic changes have far-reaching impacts on urban ecosystems, changing local microclimates and increasing environmental challenges. Special emphasis is given to The Urban Heat Island (UHI) effect, a phenomenon of temperature rise in urban areas relative to adjacent rural areas of increasing social and environmental importance [5]. For economic growth and technological progress (internationally), urbanization is a driver and a result, and is associated with different environmental problems. This challenge will lead to the extinction of biodiversity, as well as the depletion of urban green spaces and ecosystem degradation [6,7]. Additionally, when urban sprawl becomes uncontrolled, energy consumption increases; it exacerbates the changes in rainfall pattern, humidity and climate change [8]. Many environmental impacts of urbanization are striking, and perhaps the most urgent and pressing environmental problem that needs urgent scientific and policy attention is the Urban Heat Island (UHI) effect. Given that asphalt and concrete are commonly used in building materials, whose thermal properties are such that they absorb and retain heat far too efficiently compared to natural material surfaces, it makes sense that this phenomenon occurs [9]. As a result, thermal energy output capacity is more limited in metropolitan areas versus rural thermal energy [10].

It turns out that different manifestations of the UHI effect are caused at different levels (city roof level, boundary layer, surface) depending on the temperature data. As previously mentioned [11], these are referred to as canopy UHI, boundary layer UHI, and surface UHI, respectively. Nevertheless, in these case studies, surface UHI is also defined using remotely sensed land surface temperature (LST) data, particularly concerning the development of urban thermal anomalies. To address public health concerns about urban sustainability and environmental resilience [12], it is necessary to understand surface UHIs. The Urban Thermal Field Variance Index (UTFVI) is applied to quantify urban thermal field variability and its associated environmental impacts on urban areas [13,14]. Simply put, the UTFVI is in essence a means to explore urban thermal spatial forms and the connections between land use patterns. Along with remote sensing indices like Normalized Difference Vegetation Index (NDVI), Normalized Difference Water Index (NDWI), Normalized Difference Bareness Index (NDBaI) and Normalized Difference Built-Up Index (NDBI), it also offers a more precise insight into urban thermal dynamics and their surrounding factors, with reference to sustainable urban planning, particularly the Las Vegas city case, as shown by [14].

Urban expansion is the subject of extensive research in both developed and developing countries. In developed countries, studies have been conducted on advanced urban dynamics, infrastructure development, and the environmental impacts of urban sprawl [15]. However, rapid urbanization and its complex challenges are generating great interest in India and Pakistan. However, most research has focused on social and environmental impacts related to land use changes, natural resource depletion, and infrastructure pressures of urban growth in these regions [16,17,18,19,20,21,22,23]. The role of political environments, economic transformation, and technological advances in determining land use forms has been extensively studied in China, one of the most rapidly urbanizing countries in the world [24,25,26]. From the perspective of urban expansion, habitat sprawl, biodiversity, water resources, and land use systems were investigated in Mexico [27].

Considering the above literature, urbanization mainly contributes to the thermal effect on various land covers. Ceredigion County in the UK is facing increasing environmental and thermal challenges due to land use change, urbanization, and climate variability. These changes threaten biodiversity, destabilize local ecosystems, and contribute to growing thermal anomalies such as the Urban Heat Island (UHI) effect. The county is a rural area in which the environment is changing quickly and urgently needs attention. Robust tools for assessing land cover dynamics, ecological health, and surface temperature fluctuations are available using remote sensing (RS) and geographic information systems (GIS) techniques. In this study, we evaluated the thermal effects based on spectral indices (NDVI, NDBI, NDWI, NDBaI), LULC, LST, UHI, and UTFVI. This study accordingly seeks to (1) determine the temperature fluctuations in the study area, (2) evaluate the ecological assessment of the study area, and (3) monitor land use patterns in the study area. Using RS and GIS to examine Ceredigion County as an ecological and thermal assessment county is a novel study, as this area has not been previously explored. While similar methods have been used elsewhere, this work fills an important research gap by examining Ceredigion’s unique environmental and thermal dynamics. Due to the lack of prior work, this is a good place to study the changes in land use, ecological health, and thermal anomalies in the county. We prioritize the use of such advanced geospatial tools in informing sustainable planning and environmental management in the region.

### Key Contributions and Novelties of This Study

The key contributions and novelties of this study are as follows:

- **Regional Novelty:** This research presents the first comprehensive multi-decadal (2003–2023) analysis of land surface temperature (LST), urban thermal field variance index (UTFVI), and key spectral indices specifically for Ceredigion County, Wales. Unlike previous studies focused on larger urban centers such as Cardiff and Swansea, this work addresses the unique thermal dynamics and land cover transitions in a predominantly rural county with smaller urban areas, filling an important regional knowledge gap.
- **Integrated Methodological Framework:** The study introduces a novel, integrated workflow that combines high-resolution multi-temporal Landsat imagery, detailed spectral index calculations, and advanced spatial analyses in ArcGIS Pro 3.0, alongside machine learning models (Linear Regression and Random Forest) to predict LST trends up to 2030.
- **Actionable Insights for Policy and Planning:** Beyond technical contributions, the study delivers new scientific evidence that even modest urban expansion in predominantly rural areas like Ceredigion can produce significant localized urban heat island effects.

## 2. Materials and Methods

### 2.1. Study Area

**Figure 1:**
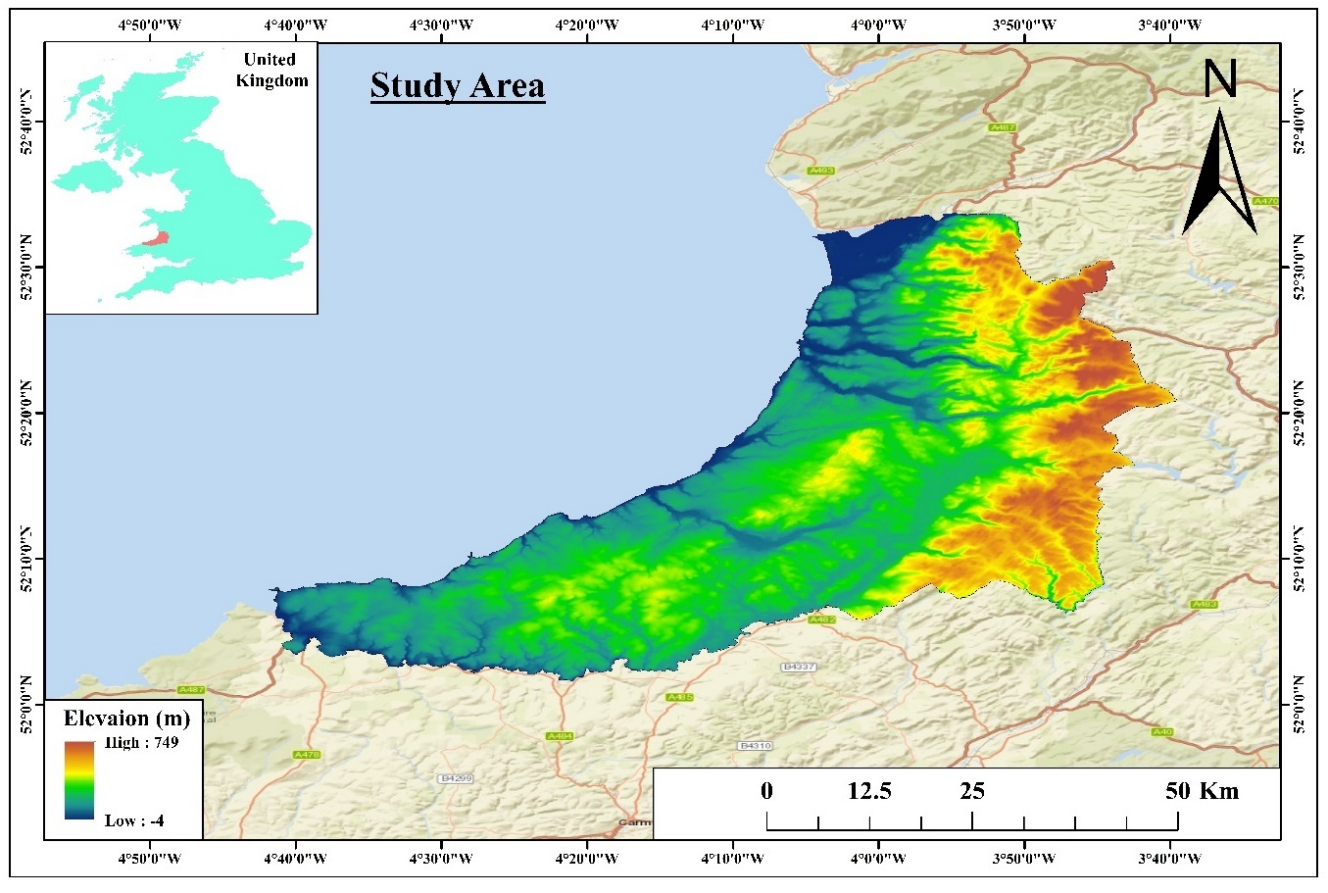
Geographical location of the study area (Ceredigion County).

Figure 1 shows Ceredigion County. It is a largely rural county located in West Wales in the United Kingdom. Divided between rolling hills, coastal cliffs, and agricultural land, the landscape is a very varied landscape with a lot of history. Ceredigion covers an area of approximately 1795 square kilometers (latitude 52.1150° N to 52.4570° N and longitude −3.9890° W to −4.7970° W). Most relevantly, the city was given as having a population of around 70,000. The town of Aberystwyth is the county capital and an important administrative and economic center. Due to its proximity to the Irish Sea, Ceredigion has a temperate maritime climate. Temperatures are mild and warm in summer, and winters are cool, with annual variations of around 5 °C to 19 °C. Summer temperatures are mostly moderate, with July and August being the warmest and January the coldest. The average seasonal fluctuations are mostly moderate. Ceredigion’s rainfall is typically high at 1000–1500 mm per year, but it is variable across the county. The rainfall in the mountainous area is higher and heavier than in the coastal area. An excellent area to study ecological and thermal patterns of land use change and environmental sustainability is in a county with such distinctive climatic and geographical characteristics.

### 2.2. Data Source

The Land Surface Temperature (LST), Urban Thermal Field Variance Index (UTFVI), and key spectral indices were calculated in this study using satellite imagery from the United States Geological Survey (USGS), specifically Landsat 5, Landsat 8, and Landsat 9 for the years 2003, 2013, and 2023. These satellite datasets were chosen to provide high temporal resolution and consistent multi-decadal observations of environmental and thermal dynamics within the study area.

All data processing, environmental metric extraction, and analyses were conducted entirely within ArcGIS Pro. Initial pre-processing steps involved radiometric and atmospheric corrections to ensure accurate surface reflectance values, as well as spectral band corrections to harmonize data across different Landsat sensors, thereby enabling reliable multi-temporal analysis. False Color Composite (RGB) images were generated to facilitate visual interpretation and aid in distinguishing various land cover classes. Supervised classification was performed using the Maximum Likelihood Classification (MLC) algorithm, guided by carefully selected training samples representative of distinct land cover types. The resulting classifications were validated through accuracy assessments, producing land use and land cover maps for each study year.

Several key spectral indices were derived from the processed imagery, including the Normalized Difference Vegetation Index (NDVI) for assessing vegetation health, the Normalized Difference Built-up Index (NDBI) for identifying urban and built-up areas, the Normalized Difference Water Index (NDWI) for detecting water bodies and moisture conditions, and the Normalized Difference Bareness Index (NDBaI) for mapping barren land surfaces. These indices were computed directly within ArcGIS Pro using established algorithms applied to the relevant spectral bands.

LST was derived using the Thermal Infrared (TIR) band, which is central to remote sensing of surface thermal properties. The TIR band captures the longwave thermal radiation naturally emitted from the Earth’s surface in the 8–14 µm wavelength range. Unlike optical or near-infrared bands that measure reflected sunlight, the TIR band senses emitted energy, making it invaluable for monitoring surface temperature independently of solar illumination. This capability enables the detection of both diurnal and nocturnal thermal patterns and supports continuous environmental monitoring under diverse weather and lighting conditions. The TIR band provides critical input for quantifying LST, a key indicator of surface energy balance and environmental stress, allowing researchers to evaluate phenomena such as urban heat islands (UHIs), land degradation, drought severity, and climate-driven thermal anomalies. In this study, the TIR data were instrumental in mapping LST patterns across various land cover types and tracking changes associated with urban expansion and land surface modifications over time.

Thermal analysis involved converting Digital Numbers (DNs) from the thermal band to Top of Atmosphere (TOA) radiance, which was subsequently transformed into Brightness Temperature. Further computations included estimating proportional vegetation cover, which informed the calculation of land surface emissivity (LSE), a critical parameter for accurately deriving LST. Using these parameters, LST was computed for each time period, facilitating detailed assessment of spatial and temporal thermal dynamics.

Based on the derived LST data, additional analyses were performed to quantify urban thermal characteristics. Urban Heat Island (UHI) intensity was assessed by comparing temperature differentials between urban areas and their surrounding environments. The Urban Thermal Field Variance Index (UTFVI) was calculated to evaluate spatial variations in thermal stress, and Thermal Comfort Zones were delineated based on specific thermal thresholds relevant to human comfort and urban sustainability. Finally, correlation analyses were conducted to explore relationships between LST and key spectral indices (NDVI, NDBI, NDWI, and NDBaI), providing insights into the interplay between land cover dynamics and urban thermal environments. The comprehensive workflow implemented in ArcGIS Pro enabled robust spatial analysis, accurate derivation of thermal and ecological metrics, and effective visualization of multi-decadal trends in the study area.

The specifications of the satellite images used in this study, including sensor types, spatial resolution, and cloud cover conditions, are summarized in Table 1. Cloud cover represents the proportion of each image obscured by clouds at the time of acquisition, which is an important factor for ensuring accurate land cover classification and thermal analysis.

To perform machine learning-based predictions of LST and spectral index dynamics for the period 2024 to 2030, the processed datasets were exported and analyzed in Python (version 3.10) within Jupyter Notebook (version 6.5.4). Predictive models, including Random Forest Regression and Linear Regression, were implemented using the scikit-learn library (version 1.2.2).

### 2.3. Data Analysis

#### Land Use and Land Cover

**Figure 2:**
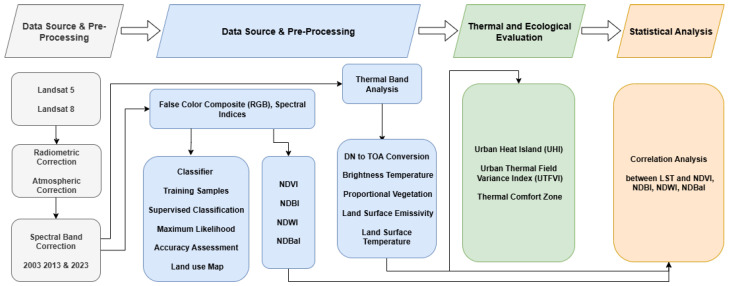
Methodological framework of study.

A novel method of automatic land cover type classification of Landsat images, to remedy the spectral heterogeneity problem among different land cover types, was developed [28]. A hybrid combination of unsupervised and supervised classification methods was adopted to improve the accuracy [29,30]. All Landsat images were grouped into ten different land use and land cover categories, including shrubland, forest, swamp, agriculture, residential, rangeland, water, open space, and runways. Delineation of polygons around regions of interest was used to reduce spectral overlap and enable precise mapping. LULC categories were visualized, and 60 spectral signatures were created for them. We performed supervised classification using the Maximum Likelihood Classification (MLC) algorithm and performed a hybrid classification comparison with higher-resolution images. Post-classification refinements to address mixed pixel issues encountered in the visual interpretation of the duck resulted in improved accuracy. Land use and land cover transition (LULC), such as from agricultural to another educational or natural area, was quantified by overlay analysis in ArcGIS. By relying on digital elevation maps and high-resolution satellite imagery, the SD team was able to correct misclassified pixels. The NDVI uses a systematic approach that removes the ambiguity of the science inputs from these outputs. We assessed classification reliability using the kappa coefficient for classification reliability with ground truth data and stratified random sampling (Figure 2). By applying this systematic approach, dynamic land cover changes were mapped and monitored with high resolution to enhance understanding of LULC transitions in time.

### 2.4. Land Surface Temperature (LST)

A critical analysis of land surface thermal variations requires accurate estimation of Land Surface Temperature (LST), which is a key indicator in understanding urban heat dynamics, vegetation health, and land cover change. In this study, LST was retrieved from Landsat 5, 8 and 9 Thermal Infrared Sensor (TIRS) Bands using the Radiative Transfer Equation (RTE). This method incorporates both surface emissivity and atmospheric corrections, ensuring higher accuracy in temperature estimation compared to simplified mono-window or single-channel algorithms. The retrieval process involved the following standard steps:

#### 2.4.1. Step 1: Conversion of Digital Numbers (DNs) to Top-of-Atmosphere (TOA) Radiance

The initial step involves converting the raw digital number (DN) values of the thermal band to TOA spectral radiance using the rescaling equation provided in the Landsat metadata, as described in [31]:

(1) $$L_{\lambda }=M_{L}\times Q_{\mathrm{cal}}+A_{L}$$

where: $L_{\lambda }$ = TOA spectral radiance (W/m^2^/sr/μm); $M_{L}$ = Radiance multiplicative scaling factor (from metadata); $A_{L}$ = Radiance additive scaling factor (from metadata); $Q_{\mathrm{cal}}$ = Calibrated DN values of the thermal band.

#### 2.4.2. Step 2: Conversion of Radiance to Brightness Temperature (BT)

The radiance is then converted to Brightness Temperature (BT), representing the effective temperature of a black body that emits the same radiance at a specific wavelength. This is calculated using the thermal constants $K_{1}$ and $K_{2}$ from the Landsat metadata [31]:

(2) $$BT=\frac{K_{2}}{\ln\left(\frac{K_{1}}{L_{\lambda }}+1\right)}-273.15$$

where: BT = Brightness temperature in °C; $K_{1}$, $K_{2}$ = Prelaunch calibration constants specific to the sensor. 273.15 is subtracted to convert the temperature from Kelvin to Celsius.

#### 2.4.3. Step 3: Estimation of Land Surface Emissivity (LSE)

Surface emissivity (ε) accounts for the difference between the ideal black body and the actual land surface emissivity. It is computed using the NDVI-based approach as proposed by [32]:

##### NDVI Calculation:


(3) $$NDVI=\frac{NIR-Red}{NIR+Red}$$

##### Proportion of Vegetation (PV):


(4) $$PV={\left(\frac{NDVI-NDVI_{min}}{NDVI_{max}-NDVI_{min}}\right)}^{2}$$

##### Emissivity Estimation:


(5) ε=0.004×PV+0.986

This method provides a pixel-wise emissivity value based on vegetation cover fraction and is particularly suitable for heterogeneous urban and semi-natural landscapes.

#### 2.4.4. Step 4: Atmospheric Correction and LST Retrieval

To obtain accurate land surface temperature, atmospheric effects must be corrected. This is done using the Radiative Transfer Equation (RTE), which considers surface emissivity, atmospheric transmittance, and upwelling/downwelling radiance components [33]:

(6) $$LST=\frac{BT-\left(1-\varepsilon \right)\cdot \left(L_{↓}+L_{↑}\right)/\varepsilon }{\tau }$$

where: τ = Atmospheric transmittance; $L_{↑}$ = Upwelling radiance (W/m^2^/sr/μm); $L_{↓}$ = Downwelling radiance (W/m^2^/sr/μm);

The atmospheric parameters (τ, $L_{↑}$, $L_{↓}$) are typically obtained using atmospheric correction tools, based on image acquisition date, time, and location. This approach yields more reliable LST estimates, especially in climatologically variable regions.

#### 2.4.5. Spectral Indices

NDVI, NDBI, NDWI, and NDBaI were used in this study to analyze the changes in LULC concerning LST. The NDVI [30], which is used as the most practical indicator of vegetation density, was examined for vegetation cover. Equation (7) was used to calculate the NDVI.

(7) $$NDVI=\;\frac{NIR-Red}{NIR+Red}$$

One of the built-up indices commonly used in studies of urban land build-up data is the NDBI [30,34]. It displays the density of buildings in the urban area. To compute it, Equation (8), proposed by [30,35], is used.

(8) $$NDBI=\;\frac{SWIR-NIR}{SWIR+NIR}$$

Water bodies can be defined and extracted from an area using NDWI, which is more appropriate than other water indices. The NDWI Equation (9) was put forth to calculate NDWI from the green and SWIR bands’ surface reflectance data [30].

(9) $$NDWI=\;\frac{Green-SWIR}{Green+SWIR}$$

Bareness monitoring was performed through NDBaI. Equation (10) calculates the NDBaI with the use of SWIR and TIR bands [30].

(10) $$NDBaI=\;\frac{SWIR-TIR}{SWIR+TIR}$$

An Urban Heat Island (UHI) has different atmospheric conditions, and annual variations make it impossible to compare images taken in different years. To compare the images equally, Equation (11) is utilized. They were all created by [36].

(11) $$UHI=\;\frac{LST-LST\;mean}{SD}$$

Urban Thermal Field Variance Index (UTFVI) is a region where the normalized LST values are 1 °C higher than the mean LST by 1° higher than 1. Numerous investigations [36] have been used to ascertain whether UTFVI exists in a study area. Equation (12) attempts to offer a numerical evaluation of UTFVI [4].

(12) $$UTFVI=\;\frac{LST-LST\;mean}{LST\;mean}$$

## 3. Results

### 3.1. Assessment of Land Use

**Figure 3:**
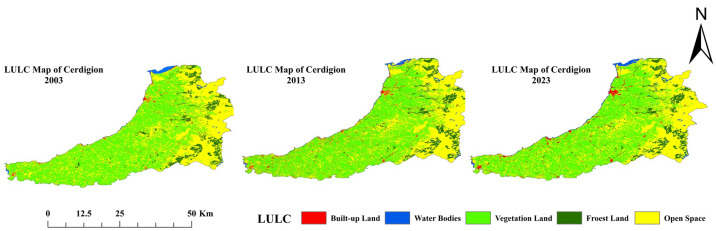
Land use and land cover map of three epochs: 2003, 2013, and 2023 (Source: Author).

Land use and land cover (LULC) data for 2003, 2013, and 2023 indicate significant land use and landscape pattern changes over the two decades. The land area in the urban environment has grown, increasing from 20 km^2^ in 2003 to 62 km^2^ in 2013 and further to 105 km^2^ in 2023 (Figure 3). This rapid urbanization and population growth, as well as the expansion of infrastructure, are reflected in this sharp increase. This was likely caused by the conversion of other land types, particularly agricultural land. In 2003, the agrarian area was 954 km^2^; in 2013, it was 938 km^2^; and in 2023, it will be 893 km^2^. The decline may be due to the effects of urban sprawl, new agricultural practices, or land degradation. The forest area is also becoming even smaller, decreasing from 129 km^2^ in 2003 to 120 km^2^ in 2013 and 103 km^2^ in 2023. There are several reasons for the loss of forest cover, such as deforestation for construction or agricultural purposes, or for industrial use, threatening biodiversity and climate regulation. The water areas have increased from 22 km^2^ in 2003 to 35 km^2^ in 2013 and then to 42 km^2^ in 2023. However, this growth could be due to better water management policies, the construction of dams, or natural events such as increased rainfall. The open space fluctuation decreased from 881 km^2^ in 2003 to 851 km^2^ in 2013 and then recovered slightly to 863 km^2^ in 2023 Table 2. Open space refers to areas with sparse vegetation, grasslands, scrublands, or bare soil that are neither built-up, forested, nor water bodies. This could be due to urban planning and conservation efforts or to changes in land restoration techniques. This panel highlights the dynamic nature of the connection between human activities and natural landscapes and highlights the importance of sustainable land management approaches. The accuracy of LULC is discussed in Table 3. Accuracy assessment was performed using reference data from the UKCEH Land Cover Maps [37], supplemented with high-resolution imagery from Google Earth and Sentinel-2. These sources ensured robust validation across all study years (2003, 2013, and 2023).

### 3.2. Assessment of Surface Temperature

**Figure 4:**
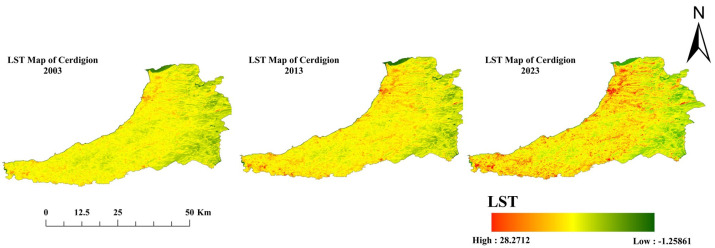
Land surface temperature map of three epochs: 2003, 2013, and 2023 (Source: Author).

Figure 4 illustrates changes in land surface temperature (LST) in Ceredigion County over the last two decades, covering the years 2003, 2013, and 2023. The data reveal a clear upward trend in temperature extremes, reflecting the combined influences of climate change, urban expansion, and land use change. In 2003, the county-wide mean LST was approximately 21.4 °C, with a maximum of 26.0 °C and a minimum of 17.0 °C (see Table 4). By 2013, the mean had increased to 22.5 °C, and the maximum reached 27.0 °C, indicating a gradual warming trend. The most significant rise occurred by 2023, when the county-wide mean LST reached 23.65 °C, while urban areas averaged as high as 27.1 °C, and maximum county-wide temperatures peaked at 28.27 °C.

These changes are consistent with previous studies reporting urban heat island intensification and rising LST in urban and peri-urban regions. Elevated LSTs can exacerbate heat waves, disrupt ecosystems, and affect agricultural productivity due to increased thermal stress. Furthermore, sustained high temperatures contribute to urban heat island effects, reduce biodiversity, and pose challenges for sustainable land management. Overall, the observed trends underscore the urgent need for climate change mitigation, sustainable urban planning, and adaptation strategies to protect both human and ecological well-being.

### 3.3. Spectral Index Calculation

**Figure 5:**
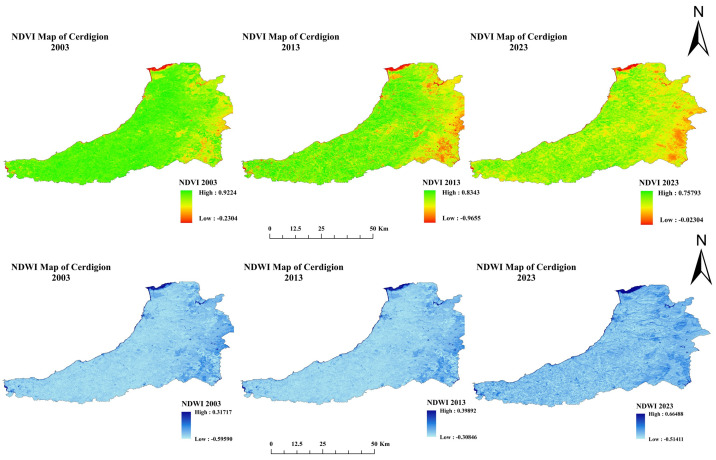
NDVI and NDWI map of three epochs: 2003, 2013, and 2023 (Source: Author).

The NDVI for the selected years from 2003 to 2023 is summarized as an overview of the changes in the Normalized Difference Vegetation Index (NDVI) from 2003 to 2023, with high and low NDVI values highlighted (Figure 5). A commonly used metric is the NDVI to measure vegetation health, density, and greenness, with positive values indicating healthy vegetation and sparse or non-vegetated landscapes having low or negative values. The 2003 NDVI value was high (0.9224), indicating dense and healthy vegetation in some areas. The low NDVI value was −0.2304, indicating regions with minimal or no vegetation cover. The high NDVI value of 0.9602 a decade later, in 2013, suggests that vegetation health or density has declined. At the same time, the low NDVI decreased significantly to −0.9655, which may be due to an increased rate of stroke or land degradation. The high NDVI value further decreased from 0.919774 to 0.75793 by 2023, indicating continued vegetation degradation over the two decades. Nevertheless, the low NDVI value regained mobility and rose to −0.02304, indicating some recovery or transformation of formerly degraded or barren areas. Factors such as deforestation, urban expansion, climate change, and agricultural intensification may have hurt vegetation health. These trends could be attributed to these factors. Low NDVI values could partially recover by 2023, particularly if reforestation efforts, land reclamation, or changes in land use practices are undertaken. The results are in line with [38]. Overall, the data highlights the importance of nature and vegetation management for a balanced ecosystem to combat vegetation loss in the context of ongoing environmental and anthropogenic pressures.

The Normalized Difference Water Index (NDWI) for 2003, 2013, and 2023 is shown in the figure, with high and low values indicated (see Figure 5). This is one of the most important metrics for monitoring water content in vegetation and observing bodies of water. High values indicate higher water availability, while lower values indicate dry or water-poor weather. Overall, the high NDWI value of 0.31717 in 2003 indicates moderate water availability in some regions. However, the significant areas characterized by water stress or drought are indicated by the low NDWI value of −0.5959. By 2013, the high NDWI increased to 0.39892, indicating increased water availability. We hypothesize that this could be due to changes in rainfall patterns, land management practices, or vegetation recovery. However, the low NDWI increased to −0.30846 at the same time, corresponding to less water-vulnerable areas. We observed the highest NDWI value shift in 2023 when improved water availability was indicated by a high NDWI value of 0.66484 in many regions. These improvements were most likely due to conservation efforts, reforestation, or more favorable climatic conditions. On the other hand, the NDWI value fell to a low of −0.51411, which was slightly lower than in 2013 but higher than in 2003. The results are in line with [39]. Overall, the situation has improved, but not all areas are affected by water shortages. The data show that water availability is a dynamic condition that depends on climatic factors and environmental management. These trends highlight the need for integrated water resource management and sustainable land use to address water scarcity and ensure ecological resilience to climate change.

**Figure 6:**
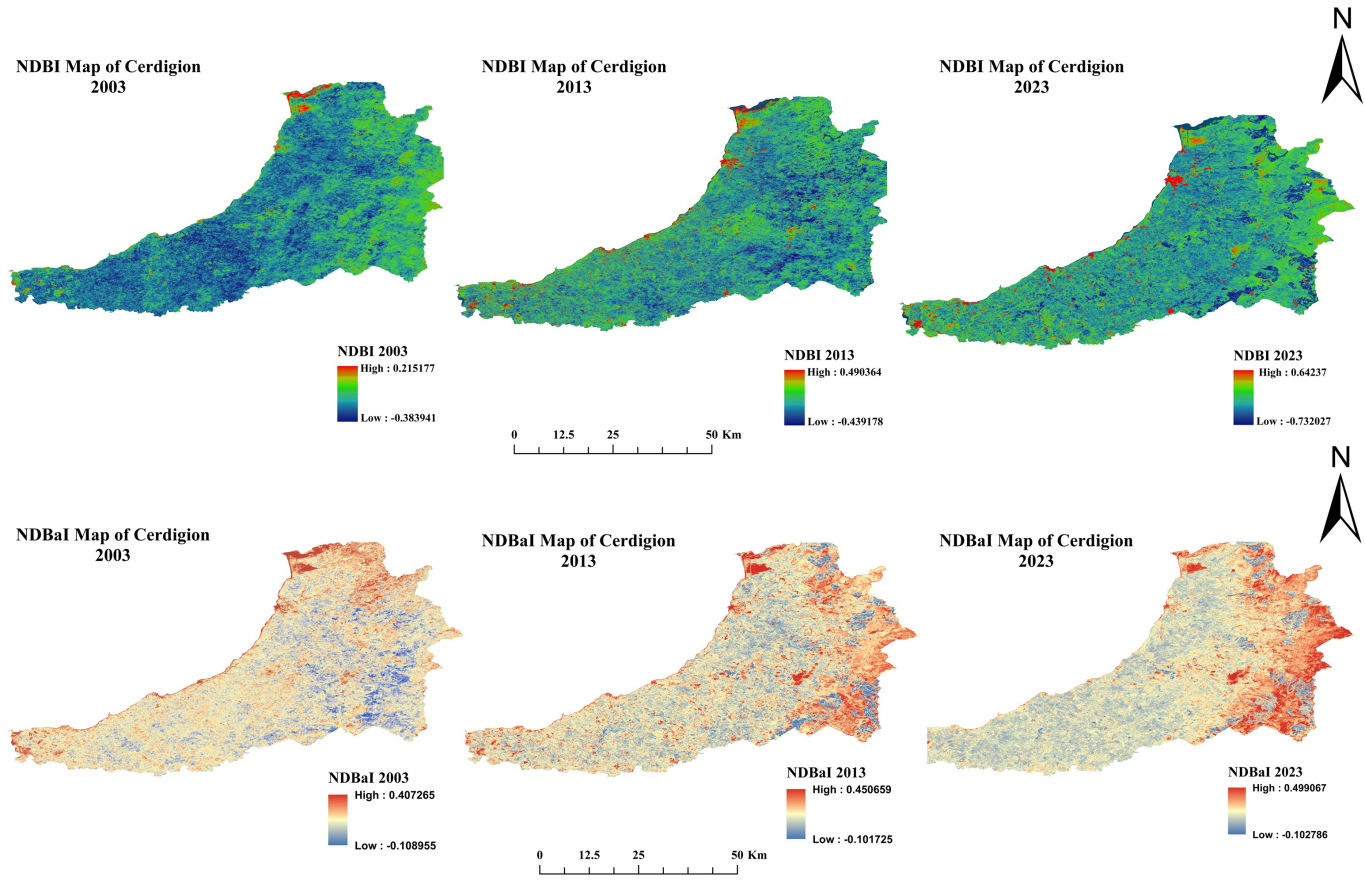
NDBI and NDBaI map of three epochs: 2003, 2013, and 2023.

NDBI values in 2003, 2013, and 2023 show changes in urbanization and development patterns over time. Urban growth is assessed using the NDBI, a key indicator (see Figure 6). Higher NDBI values indicate areas where built-up areas dominate, while lower or negative NDBI values are due to natural land cover or sparse urbanization. The peak value of high NDBI was 0.215167 in 2003, indicating that there is only moderate development in some locations. The low value of −0.383941 indicates that natural landscapes or non-urbanized regions would dominate. By 2013, the high NDBI value increased significantly over time to 0.490364, a growth rate that implies a rapid increase in urban and built-up areas over the next decade. However, the low NDBI value fell slightly to −0.439178, indicating that development spread to less urbanized and natural regions. By 2023, it reached an NDBI value of 0.64237, indicating further urban growth and densification of built-up areas. This was reflected in the low NDBI value, which dropped significantly to −0.732027 between the highly urbanized zones and the natural cover zones, where the natural land cover is maintained under the pressure of urbanization expansion. The results are in line with [40]. These trends characterize the impacts of urbanization on landscapes, amplified by population growth, economic development, and infrastructure expansion. Urban growth enables socio-economic development but also brings with it problems of habitat loss, environmental degradation, and resource strain. Such planning for sustainable urban development and land use management is crucial to finding an optimal balance between development and environmental protection and enabling future livable cities.

Figure 6 shows NDBaI values for the years 2003, 2013, and 2023 with changes in uncovered ground area over time. Monitoring soil degradation, soil exposure and the proportion of bare (infertile) areas (NDBaI) is an important metric. A positive or high NDBaI indicates the level of bare soil, while low or negative values indicate vegetation cover or water. The high (0.407265) and low (−0.108955) NDBaI values in 2003 reflect moderate to low levels of exposed soil and the presence of vegetation and water, respectively, in some locations. The value of high NDBaI increased over the next decade from 0.362110 in 2003 to 0.450659 during the period between 2003 and 2013, possibly due to deforestation, land use change or agricultural expansion. However, the low value hardly fluctuated and even improved slightly to −0.101725, indicating relative stability in natural cover regions. The high NDBaI value also increased from 0.251630 in 2023 to 0.499067 in 2023, indicating that the further expansion of bare ground areas continued in 2023. This may be due to the process of intensified urban development, soil erosion, or excessive exploitation of land resources. While the low value remained almost the same (−0.102786), it means that regions with vegetation or water did not experience major changes during this period. The results are in line with [41]. The trends show an increasing land degradation problem and increasing soil pollution situation that requires sustainable land management practices. Reforestation, soil conservation, and responsible land use planning are important initiatives to counteract soil degradation and strengthen ecological resilience.

### 3.4. Assessment of Urban Heat Island

**Figure 7:**
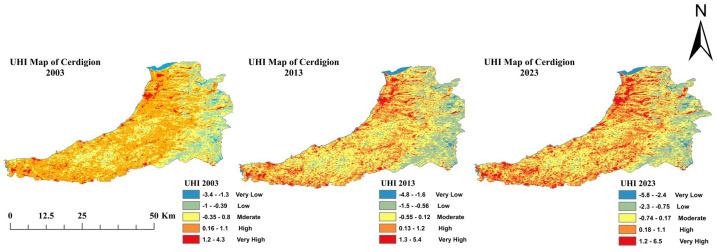
UHI map of three epochs: 2003, 2013, and 2023 (Source: Author).

Figure 7 shows the spatial distribution of urban heat island (UHI) intensity across five classes—very low, low, moderate, high, and very high—for the years 2003, 2013, and 2023 (see Table 5). The data illustrate changing UHI impacts over the past two decades as a result of urban expansion, land use change, and climate dynamics. The spatial extent of UHI in the Very Low category was 96 km^2^ in 2003, and the low and medium UHI intensity was 621 km^2^ and 456 km^2^, respectively. The largest area was covered with 732 km^2^ of high UHI intensity and 101 km^2^ of very high intensity. This distribution shows that urban areas were already well within the High and Very High categories of this heat intensity distribution. By 2013, cooler urban spaces had shrunk to just 55 km^2^… meaning the Very Low category was reduced to 55 km^2^ by 2013. Only the Low category increased slightly (633 km^2^), while Moderate UHI increased to 463 km^2^. UHI intensity increased slightly to 741 km^2^ for the High category and 114 km^2^ for the Very High category. Such a shift is likely a reflection of increasing urbanization and the greater impact of UHI. The Very Low category partially recovered to 67 km^2^ in 2023, while the Low category fell significantly to 491 km^2^. The moderate UHI saw a notable increase from 564 km^2^ to 759 km^2^. For the High and Very High categories, they increased to 759 km^2^ and 125 km^2^, respectively. These data highlight that UHI impacts are an urgent issue and should be mitigated through sustainable urban planning. Solutions include green infrastructure, urban forestry, and reflective building materials to reduce heat intensity and increase urban resilience.

### 3.5. Assessment of Thermal Conditions

**Figure 8:**
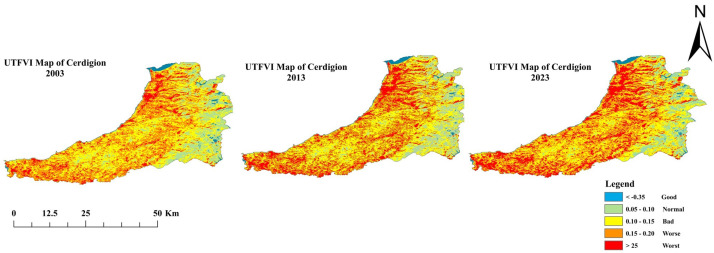
UTFVI map of three epochs: 2003, 2013, and 2023 (Source: Author).

The spatial distribution of the Urban Thermal Field Variance Index (UTFVI) in km^2^ across five categories: Good, Normal, Poor, Bad, and Worst, for the years 2003, 2013, and 2023, is shown in Figure 8. The thermal comfort and ecological qualities of urban spaces are assessed using UTFVI, a measure that indicates whether conditions are good (best) or worse (worst) (Table 6). In 2003, 151 km^2^ of urban areas had a good UTFVI as a measure of limited areas with favorable thermal conditions. The normal area was 413 km^2^ and the poor area was the largest area at 736 km^2^, which included a large proportion of urban areas with moderate thermal stress. High thermal complaints, as addressed in the Poor and Worst categories, occupied 558 km^2^ and 148 km^2^, respectively. By 2013, urbanization and heat intensity led to a shrinkage of the good area to 135 km^2^, which still represented a decline in thermally comfortable areas. This Normal category grew slightly to 446 km^2^ and the ‘Poor’ category increased to 751 km^2^. The area of the Worst and Worst categories increased to 604 km^2^ and 170 km^2^, respectively, indicating an increase the thermal stress in urban landscapes. The Good UTFVI dropped to 107 km^2^ in 2023 and the Normal plummeted to 313 km^2^. There were 781 km^2^ of Poor, 613 km^2^ of Poor, and 192 km^2^ of Poor, representing an increase in thermal conditions. The data highlight the need for urban interventions, including increasing green spaces, redesigning buildings, and cooling strategies, to reduce thermal loads and promote quality of life in the city.

### 3.6. Correlation Analysis

Correlations of LST in 2003, 2013, and 2023 are shown with spectral indices (NDVI, NDBI, NDWI, and NDBaI). The correlations presented in these equations provide clues about how surface temperature relates to other land use characteristics. The correlation coefficient between LST and NDVI (−0.013) is very weak and negative, meaning that vegetation had a very small inverse effect on surface temperature in 2003. Likewise, there is a weak and positive correlation between LST and NDWI (0.008), indicating that changes in surface temperature due to water bodies did not make a significant contribution. There is a slightly stronger positive correlation between LST and NDBI (0.017) as built-up areas have a slight influence on higher surface temperatures. However, the correlation between LST and NDBaI (0.030) is the strongest among the indices, suggesting that barren land had a noticeable but weak influence on surface temperatures (Table 7). It is observed that the correlation between LST and NDVI (−0.031) is weak and negative and there is a slight tendency for vegetated areas to reduce the surface temperature. The correlation between LST and NDBI (0.013, positive) was also weak, indicating that built-up areas did not influence surface temperature. The correlation between LST and NDWI (0.407) was quite moderately positive, which may indicate that surface temperatures were higher in 2013 because the LST was closer to the water bodies in 2013 (urban heat island effect). The correlation between LST and NDBaI (0.017) is weak and the final LST value correlates minimally with barren land (see Table 8). The negative and weak correlation between LST and NDVI of −0.031 indicates that vegetation does not make a significant contribution to moderating surface temperature. Like the correlation between LST and NDBI (0.017), it is weak and positive, meaning that built-up areas have little influence on temperature. There is a slight positive relationship between LST and NDWI (0.407), and this shows continuity between water bodies and increased surface temperatures and heat islands due to water supply. Compared to previous years, our correlation between LST and NDBaI (0.072) has increased, indicating a growing influence of barren land on surface temperatures. The results suggest that surface temperature will be more gradually affected by land use changes over the period 2003 to 2023, requiring sustainable urban planning to prevent temperature increases (see Table 9).

### 3.7. Machine Learning-Based Prediction of LST and Remote Sensing Indices (2024–2030)

#### 3.7.1. Methodology and Model Framework

To estimate the future dynamics of Land Surface Temperature (LST) and related spectral indices, two machine learning models were developed and compared: Linear Regression (LR) and Random Forest Regression (RF). The models utilized the following spectral indices as predictive features, all derived from satellite-based remote sensing:

- Normalized Difference Vegetation Index (NDVI): Indicator of vegetation health and density.
- Normalized Difference Water Index (NDWI): Proxy for surface moisture and water features.
- Normalized Difference Built-up Index (NDBI): Reflects impervious surface expansion.
- Normalized Difference Bareness Index (NDBaI): Captures bare land and exposed surfaces.

The Linear Regression model was based on the following equation:

(13) $$\hat{y}=\beta_{0}+\beta_{1}\cdot \mathrm{NDVI}+\beta_{2}\cdot \mathrm{NDWI}+\beta_{3}\cdot \mathrm{NDBI}+\beta_{4}\cdot \mathrm{NDBaI}$$

where $\hat{y}$ is the predicted LST, and $\beta_{0}$ to $\beta_{4}$ are the model coefficients derived from training on historical data (2003–2023).

The Random Forest Regression model, in contrast, is a non-parametric ensemble approach that constructs multiple decision trees during training and outputs the average prediction from all trees. This allows the RF model to capture complex, nonlinear interactions among variables and to model feature interactions more robustly.

#### 3.7.2. Prediction Results and Analysis

**Figure 9:**
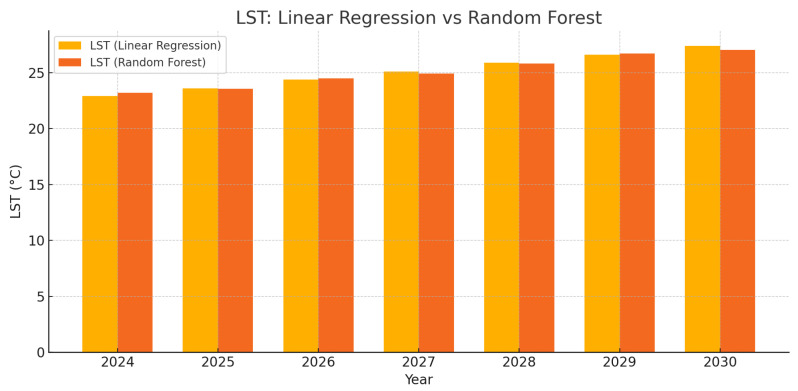
Comparison of predicted LST values using Linear Regression and Random Forest for 2024–2030.

Figure 9 shows the forecasted land surface temperature (LST) values from 2024 to 2030, derived using both Linear Regression (LR) and Random Forest (RF) models, which consistently indicate an upward trend reflecting ongoing urbanization and environmental changes in Ceredigion. The LR model projects a steady linear increase, reaching approximately 27.4 °C by 2030 in urban built-up areas, while the RF model estimates a slightly lower value around 26.8 °C, reflecting its capacity to moderate extremes through modeling non-linear interactions. These urban LST projections are realistic when compared to local climate data. For instance, the observed average maximum summer air temperature at Gogerddan for 1991–2020 ranges from approximately 18.0 °C to 19.5 °C, with projected increases to around 18.18 °C to 19.64 °C by 2030. Urban surfaces in the UK typically exhibit an additional warming of about 5 °C to 12 °C above air temperature due to the urban heat island effect [2]. Applying a mid-range urban surface difference of approximately 8.5 °C to projected summer air temperatures yields urban LST values between roughly 26.8 °C and 27.4 °C, which aligns well with the model outputs. By contrast, the county-wide average LST, which reflects Ceredigion’s predominantly rural landscape comprising approximately 7.5–8% urban areas, around 90% vegetated or rural land, and about 2% inland water bodies, is computed to be around 23.0 °C to 23.5 °C in summer. This estimate is based on a weighted average LST–air temperature difference of approximately 3.8 °C to 3.9 °C for the entire county, taking into account typical surface–air differences of 2 °C to 5 °C over vegetated areas and about 0 °C to 3 °C over water bodies. Thus, while county-wide average LST remains moderate, the higher urban values predicted by both models are scientifically consistent with local conditions and the documented thermal behavior of urban surfaces in Ceredigion.

Table 10 shows observed maximum summer air temperatures at Gogerddan Station from UK Met Office records [42], alongside computed projections for 2001–2030. Forecast trends for 2024–2030 were compared against the historical annual rate of change in air temperature computed from Gogerddan Station data, ensuring consistency between model outputs and observed regional warming patterns. County-average LST values were estimated by adding a surface–air temperature difference of 3.9 °C, reflecting Ceredigion’s land cover. The results are consistent with the LST trends predicted by this study’s regression models, confirming alignment between observed ground data and model-based forecasts.

**Figure 10:**
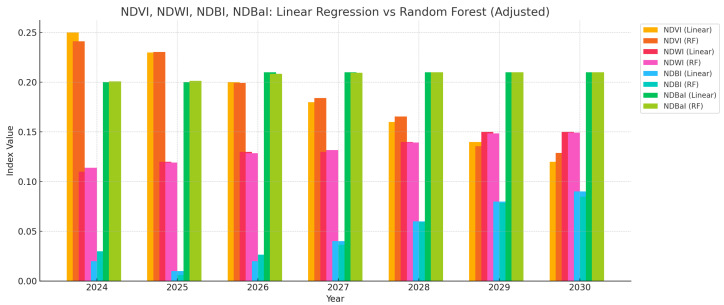
Predicted values of NDVI, NDWI, NDBI, and NDBaI from 2024 to 2030 using Linear Regression and Random Forest models.

Figure 10 compares the predicted values of NDVI, NDWI, NDBI, and NDBaI across the same period. Overall, both models produced similar patterns with the following notable trends:

- NDVI: Gradual decline, indicating vegetation loss, likely due to urban expansion and land use intensification.
- NDWI: Mild increase, possibly reflecting changes in soil moisture or impervious surfaces’ water retention.
- NDBI: Increasing values, representing the growth of built-up areas over time.
- NDBaI: Slightly rising or stable values, suggesting sustained bare surface exposure, potentially due to deforestation or land degradation.

The alignment between the two models in spectral indices further supports the general predictability of these environmental patterns using historical data. However, Random Forest consistently delivered smoother predictions and captured subtleties in temporal trends more effectively.

#### 3.7.3. Model Evaluation

Both models were quantitatively evaluated using three common metrics: the coefficient of determination ($R^{2}$), mean absolute error (MAE), and root mean square error (RMSE). The results are presented in Table 11.

The Random Forest model significantly outperformed the linear model across all metrics, indicating its superior capability in capturing nonlinearities and minimizing prediction errors. Its ability to handle complex interdependencies among features makes it a strong candidate for future remote sensing-based climate predictions in urban environments.

## 4. Discussion

Analysis of land use and land cover (LULC) data from 2003, 2013, and 2023 revealed significant changes in landscape patterns in Ceredigion County. Urban areas expanded notably from about 20 km^2^ in 2003 to 105 km^2^ in 2023, largely at the expense of agricultural and forested land. This trend mirrors broader urbanization observed elsewhere, where built-up areas increasingly encroach on natural landscapes [1,2]. However, our study uniquely focuses on a predominantly rural Welsh county, offering new insights into land cover change in less urbanized regions.

Our LST analysis shows the county-wide mean summer LST increased from 21.4 °C in 2003 to 23.65 °C in 2023. Urban areas consistently recorded higher temperatures, averaging about 27.1 °C in 2023. These findings highlight urban heat island (UHI) effects, even in moderately urbanized areas like Ceredigion. The urban–rural LST differences of roughly 3.5–4.0 °C align with previously reported ranges of 5–12 °C for UK cities [5], demonstrating that even modest urban expansion can produce significant local thermal anomalies [3].

Spectral indices such as NDVI, NDWI, NDBI, and NDBaI showed variable relationships with LST. The negative correlation between NDVI and LST underscores vegetation’s cooling role, consistent with earlier research [22]. These correlations were generally moderate to weak, suggesting that factors like urban materials and landscape structure also significantly influence surface temperatures.

The Urban Thermal Field Variance Index (UTFVI) highlighted zones of thermal stress, particularly in growing urban areas, indicating localized heat risks. This agrees with previous findings that urban expansion not only raises mean surface temperatures but also increases spatial variability [7]. Our machine learning forecasts suggest urban LSTs could reach around 27.4 °C by 2030. While based on limited time points, these projections align with regional warming trends and observed air temperature data from Gogerddan Station, supporting their plausibility.

Overall, while urban growth in Ceredigion is less intense than in larger cities, our results show that even moderate expansion in rural areas can substantially alter thermal conditions and land cover. This underscores the importance of sustainable urban planning and climate adaptation measures, as emphasized in previous research [1,7], to balance development with environmental sustainability in rural regions.

## 5. Conclusions

In conclusion, urbanization remains a powerful driver of landscape transformation worldwide, bringing significant socio-economic benefits but also creating substantial environmental challenges. As urban areas expand, natural landscapes are increasingly replaced by impervious surfaces, intensifying issues such as the Urban Heat Island (UHI) effect, elevated urban temperatures, and environmental degradation.

Our analysis of land use and land cover (LULC) changes in Ceredigion County from 2003 to 2023 reveals rapid urban growth, often occurring at the expense of agricultural and forested lands. This expansion has contributed to vegetation loss and altered water availability, underscoring the need for effective resource management strategies. Our LST analysis, alongside spectral indices (NDVI, NDBI, NDWI, and NDBaI), confirms accelerating urbanization trends and highlights increasing thermal stress. The Urban Thermal Field Variance Index (UTFVI) further demonstrates growing areas of thermal discomfort, signaling the urgency for climate adaptation and sustainable urban planning.

While the relationships between LST and spectral indices observed in this study are moderate to weak, they suggest that the thermal impacts of urbanization are influenced by complex interactions among vegetation, built surfaces, and water bodies, leading to spatial variability in thermal conditions. Addressing these challenges requires balancing economic development with environmental sustainability through strategies such as reforestation, green infrastructure, and climate adaptation measures to mitigate the negative impacts of urbanization.

Finally, although the methodology presented in this study—including multi-temporal satellite analysis and machine learning modeling—is broadly applicable and can be adapted to other regions, its transferability is subject to certain limitations. Local factors such as climate, land cover diversity, urban morphology, and data availability may influence the accuracy and reliability of results. Therefore, regional calibration and validation are essential when applying these methods to different geographical contexts.

Overall, our findings contribute valuable insights for policymakers and urban planners seeking to manage urban growth sustainably, ensuring resilient and livable environments even in predominantly rural regions like Ceredigion.

## Figures and Tables

**Table 1:** The specifications of satellite images.

Year	Satellite	Sensor	Resolution	Cloud Cover
2003	Landsat 5	TM Sensor	30 m	0.4
2013	Landsat 8	OLI and TIR Sensors	30 m	0.1
2023	Landsat 9	OLI and TIR Sensors	30 m	0.2

**Table 2:** Area of different land use in km^2^.

Year	Built-Up Land	Agriculture Land	Forest Land	Water Bodies	Open Space
2003	20	954	129	22	881
2013	62	938	120	35	851
2023	105	893	103	42	863

**Table 3:** The accuracy of LULC classification in %.

Year	User Accuracy	Producer Accuracy	Overall Accuracy	Kappa Coefficient
2003	92	93	94	93
2013	96	95	96	96
2023	95	96	96	94

**Table 4:** Descriptive statistics of summer-season LST in Ceredigion County, showing county-wide and urban averages.

Year	County Mean	Urban Mean	Max	Min	SD
	(°C)	(°C)	(°C)	(°C)	(°C)
2003	21.4	25.0	26.0	17.0	2.0
2013	22.5	26.0	27.0	18.0	2.1
2023	23.65	27.1	28.27	19.0	2.3

**Table 5:** The area in km^2^ of UHI zones.

Year	Very Low	Low	Moderate	High	Very High
2003	96	621	456	732	101
2013	55	633	463	741	114
2023	67	491	564	759	125

**Table 6:** The area in km^2^ of thermal conditions.

Year	Good	Normal	Bad	Worse	Worst
2003	151	413	736	558	148
2013	135	446	751	604	170
2023	107	313	781	613	192

**Table 7:** The correlation between LST and spectral indices of the year 2003.

	LST_2003	NDVI_2003	NDBI_2003	NDWI_2003	NDBaI_2003
**LST_2003**	1				
**NDVI_2003**	−0.01302	1			
**NDBI_2003**	0.016531	−0.47991	1		
**NDWI_2003**	0.008482	−0.94643	0.432195	1	
**NDBaI_2003**	0.029663	−0.69487	0.392459	0.606885	1

**Table 8:** The correlation between LST and spectral indices of the year 2013.

	LST_2013	NDVI_2013	NDBI_2013	NDWI_2013	NDBaI_2013
**LST_2013**	1				
**NDVI_2013**	−0.03146	1			
**NDBI_2013**	0.013265	−0.43736	1		
**NDWI_2013**	0.406936	−0.02903	0.044844	1	
**NDBaI_2013**	0.016683	−0.41392	0.952793	0.042211	1

**Table 9:** The correlation between LST and spectral indices of the year 2023.

	LST_2023	NDVI_2023	NDBI_2023	NDWI_2023	NDBaI_2023
**LST_2023**	1				
**NDVI_2023**	−0.03131	1			
**NDBI_2023**	0.016777	−0.41527	1		
**NDWI_2023**	0.406971	−0.0291	0.042179	1	
**NDBaI_2023**	0.072372	0.167893	−0.27412	0.050167	1

**Table 10:** Maximum summer air temperatures and corresponding county-average land surface temperatures (LSTs) at Gogerddan Station.

Month	1961–1990 (°C)	1971–2000 (°C)	1981–2010 (°C)	1991–2020 (°C)	2001–2030 (°C)	LST 2001–2030 (°C)
June	17.46	17.21	17.64	18.00	18.18	22.08
July	18.95	19.29	19.35	19.47	19.64	23.54
August	18.93	19.26	19.21	19.28	19.40	23.30

**Table 11:** Performance metrics of Linear Regression and Random Forest models for LST prediction.

Model	$R^{2}$ Score	MAE (°C)	RMSE (°C)
Linear Regression	0.97	0.73	0.92
Random Forest	**0.99**	**0.33**	**0.41**

## Data Availability

Data is available on request.
